# Extracorporeal Membrane Oxygenation (ECMO) Rescue Therapy in Post-cardiotomy Cardiogenic Shock: A Case Report

**DOI:** 10.2478/rjaic-2020-0019

**Published:** 2020-12-31

**Authors:** Mihai Stefan, Ovidiu Stiru, Ioana Marinica, Mihail Luchian, Alina Paunescu, Alexandra Ciurciun, Vlad Anton Iliescu, Ovidiu Chioncel, Serban Bubenek, Daniela Filipescu

**Affiliations:** 1 Institutul de Urgenta pentru Boli Cardiovasculare Prof Dr C C Iliescu Bucharest, Romania

**Keywords:** cardiogenic shock, mechanical circulatory support

## Abstract

Cardiogenic shock is a constant challenge for the intensivist when complicating a myocardial infarction, due to the high rate of associated morbidity and mortality, especially in the setting of mechanical complications such as papillary muscle rupture.

We present the case of a 49-year-old woman with cardiogenic shock due to acute myocardial infarction (AMI) complicated by severe mitral valve insufficiency due to papillary muscle rupture. She was treated initially by medical optimization, followed by mitral valve replacement and complete surgical revascularization, requiring rescue mechanical circulatory support by extracorporeal membrane oxygenation (ECMO).

ECMO proved to be a rescue therapy in a patient with refractory cardiogenic shock after urgent cardiac surgery.

## Introduction

Refractory cardiogenic shock can be defined as ongoing evidence of tissue hypoperfusion despite administration of adequate doses of two vasoactive medications and treatment of the underlying etiology. It carries a hospital mortality as high as 50%.[1] Cardiopulmonary support with veno-arterial extracorporeal membrane oxygenation (ECMO) has been proved to be a useful tool in the management of shock syndromes.[2] In cardiac surgery, in the perioperative period, ECMO is rapidly evolving as a readily available tool for rescue therapy in rapidly deteriorating patients as it offers multi-organ support and can be placed in patients with left ventricular (LV), right ventricular (RV), or bi-ventricular failure.

## Patient presentation and initial workup

A 49-year-old female was admitted directly to the intensive care unit (ICU) from another institution, after having suffered a silent myocardial infarction (MI), possibly in the previous 7 days, complicated by a papillary muscle rupture with severe mitral valve regurgitation. At admission, the patient was mechanically ventilated and showed signs of cardiogenic shock and organ dysfunction, i.e. cold, clammy skin, anuria with elevated serum creatinine, elevated serum lactate (6 mmol/l), and severe LV dysfunction (LV ejection fraction [LVEF] 30%), on transthoracic echocardiographic (TTE) examination. Standard monitoring was supplemented by an arterial line placed on the left radial artery and a pulmonary artery catheter via the right jugular vein.

## Diagnosis and management

Transesophageal echocardiography (TEE) confirmed posterior papillary muscle rupture, with severe mitral regurgitation and severe LV dysfunction (Figure 1).

Initial workup was completed with a coronary angiography, which showed triple-vessel coronary artery disease. At this point, the institutional Heart Team (including a cardiologist, cardiovascular surgeon, and anesthesiologist) decided that, given the high mortality risk (Euroscore II 31.47%, Sequential Organ Failure Assessment [SOFA] score 10), initial management should focus on stabilizing the patient, with surgery planned in the same hospitalization.

An intra-aortic balloon pump (IABP) was inserted via the right femoral artery, and the patient was optimized by multimodal treatment, including inodilator therapy with levosimendan and continuous renal replacement therapy (CRRT) with hemodiafiltration. Remission of cardiogenic shock occurred after 24 hours and renal function improved, allowing for surgical treatment on ICU day 5, while she was still on IABP. Although TTE showed no improvement of LV function, cardiac output measured by thermodilution increased from 2.1 to 2.4 l/min/m^2^.

**Figure:**
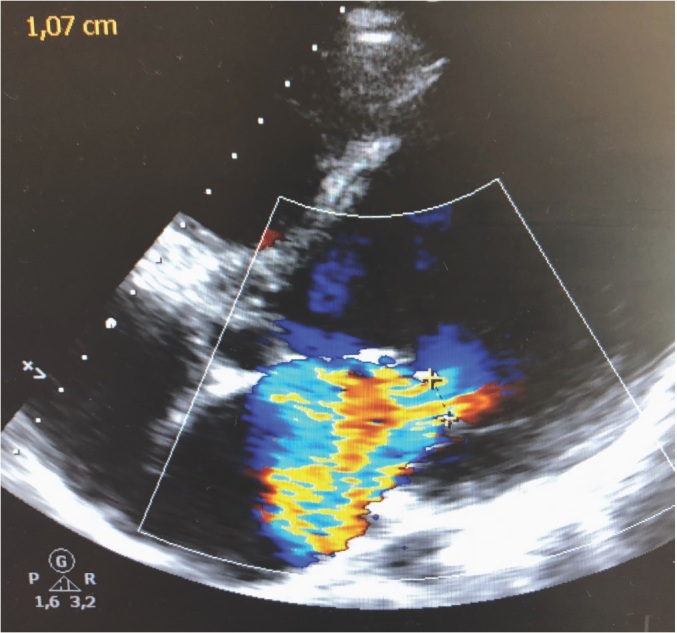
Figure 1

A triple coronary artery bypass graft (a left internal mammary artery to the left anterior descending artery and two saphenous vein grafts to the obtuse marginal branch and right coronary artery) and mitral valve replacement (size 27 Sorin Bicarbon mechanical prosthesis) was performed. The procedure was done under cardiopulmonary bypass (CPB), with modified ultrafiltration and hemoadsorption for cytokine removal. Despite complete revascularization, CPB weaning was difficult due to bi-ventricular dysfunction, but was achieved under high doses of inotropes and vasopressors (dobutamine 10 μg/kg/min and norepinephrine 0.5 μg/kg/min) and inhaled nitric oxide (NO) 20 ppm for RV failure.

Postoperatively, the patient developed cardiogenic shock (cardiac index of 1.9 l/min/m^2^) that was refractory to high doses of vasoactive drugs (dobutamine, norepinephrine, and vasopressin), as well as inhaled NO and mechanical support with IABP. The need of an advanced mechanical circulatory support was decided 24 hours postoperatively. A veno-arterial ECMO was used via a left femoro-femoral approach, while maintaining the IABP for adequate LV unloading. The ECMO device (flow of 3.8–4 l/min) was in place for 9 days. While on ECMO, the patient had neither pump-related nor cannulation-related complications, and LV ejection was maintained under low-dose inotropes, with no signs of pulmonary congestion. No further organ dysfunction developed. Postoperatively, there was no need of CRRT.

Weaning was undertaken using an ultrasound-guided protocol and IV inodilators (levosimendan) and was successful on postoperative day 10. The patient was extubated on postoperative day 12. The IABP was removed 2 days later. The TEE examination after weaning showed an LVEF of 40%, normal RV, and adequate mechanical mitral valve function. The patient was shifted to the ward on postoperative day 20 and discharged from the hospital 1 week later.

## Discussion

International guidelines recommend emergency surgery in cases presenting with mechanical complications of acute myocardial infarction (AMI), although perioperative mortality is high.[3] The risk of mortality is even higher in cases admitted with preoperative cardiogenic shock.[4] Hemodynamic stabilization is based on afterload reduction and inotropic treatment plus diuretics.[3] In our patient, IABP was used for afterload reduction and levosimendan for both inotropy and afterload reduction. As the patient had anuria from the presentation, CRRT with hemodiafiltration aimed to control the fluid, electrolytic, and metabolic balance. This strategy resulted in a reduction of the regurgitant volume and increased cardiac output, allowing for optimization of the patient’s condition preoperatively. While not routinely recommended in cardiogenic shock,[5] IABP is indicated for circulatory support in mechanical complications of AMI.[3] Levosimendan has been advocated as a useful agent in both cardiogenic shock and for weaning of veno-arterial ECMO.[6] Unfortunately, the use in patients on CRRT has an unpredictable effect on the parent agent and its metabolites, and consequently on the hemodynamics.

Despite the complete revascularization and replacement of insufficient valve, the patient developed postoperative cardiogenic shock refractory to conventional therapy. Using a stepwise approach, the next choice was to use ECMO as a rescue mechanical circulatory support and bridge to recovery. Compared to other short- and long-term circulatory assist devices, ECMO offers the advantage of both cardiac (bi-ventricular) and respiratory support.[7] Organ support was optimal, as documented by lack of new-organ dysfunction and normal serum lactate levels, and LV unloading was effective through the IABP[5] which was already in place. Cardiac function recovered slowly under ECMO, and weaning was uneventful after 9 days of circulatory support. An ultrasound-guided weaning strategy was used,[8] and the patient was discharged home in a good condition.

## Conclusions

Mechanical complications of AMI associated with cardiogenic shock represent a challenging setting for the Heart Team. Mechanical circulatory support is needed both pre- and postoperatively, and a stepwise approach is recommended. ECMO as a rescue therapy in a patient with refractory cardiogenic shock after urgent cardiac surgery, already on IABP, inotropic, vasoconstrictor support, and inhaled NO proved to be successful.
